# Research on Resource Allocation in Cognitive Radio Networks Assisted by IRS

**DOI:** 10.3390/s26030978

**Published:** 2026-02-03

**Authors:** Shuo Shang, Zhiyong Chen, Dejian Zhang, Xinran Song, Mingyue Zhou

**Affiliations:** School of Computer Science and Engineering, Changchun University of Technology, Changchun 130012, China

**Keywords:** cognitive radio, maximizing energy efficiency, intelligent reflecting surface, spider wasp optimization

## Abstract

To address the reduction in energy efficiency caused by severe signal attenuation during long-distance transmission in cognitive radio networks, this paper constructs an IRS-assisted and energy-constrained relay cognitive radio resource allocation model operating in the underlay mode. By introducing controllable reflective paths, the model enhances link quality and improves energy utilization efficiency. Our objective is to maximize the energy efficiency of secondary users while satisfying the interference constraints imposed on the primary user. To effectively solve the highly non-convex and high-dimensional optimization problem, we propose a Chaotic Spider Wasp Optimization algorithm. The algorithm employs chaotic mapping to initialize the population and enhance population diversity, and incorporates a dynamic trade-off factor to achieve an adaptive balance between hunting and nesting behaviors, thereby improving global search capability and avoiding premature convergence. In addition, the Jain fairness index is introduced to enforce fairness in the power allocation among secondary users. Simulation results demonstrate that the proposed model and optimization method significantly improve system energy efficiency and the stability of communication quality.

## 1. Introduction

With the rapid evolution of wireless communication technologies, the scarcity of spectrum resources has become increasingly severe. Cognitive radio (CR) has been proposed as a promising solution to this critical challenge [1,2]. Since a large portion of the licensed spectrum is underutilized, cognitive radio technology enables secondary users (SUs) to access unlicensed spectrum opportunistically while guaranteeing that the communication quality of primary users (PUs) is not adversely affected, thereby significantly improving spectrum utilization efficiency [3].

In long-distance transmission scenarios, severe signal attenuation makes conventional single-hop communication insufficient to meet reliability requirements. As a result, relay-assisted transmission is widely adopted to enhance communication performance. Previous studies have demonstrated that energy-harvesting relays can prolong network lifetime while improving spectrum efficiency and transmission reliability. Si et al. introduced a nonlinear energy-harvesting relay scheme and selected the relay with the strongest received power for cooperative transmission [4], thereby improving spectrum efficiency and communication performance. Boddapati et al. analyzed an incremental relaying scheme in cooperative multi-hop networks to reduce throughput loss during multi-hop transmissions [5]. Mondal et al. employed a time-switching-based energy-harvesting relay to enhance spectrum utilization and network lifetime in cognitive radio networks [6]. Extensive studies have confirmed that relay-assisted transmission plays a crucial role in extending communication coverage and improving spectrum efficiency [7,8,9].

With the growing demand for higher energy efficiency and broader coverage in wireless networks, traditional relay techniques alone are no longer sufficient to further enhance system performance. In this context, intelligent reflecting surfaces (IRSs), as a novel passive and reconfigurable transmission technology, have attracted considerable attention due to their ability to intelligently reconfigure the wireless propagation environment, thereby significantly improving link quality and energy efficiency [10,11]. Wang et al. investigated energy efficiency maximization in non-orthogonal multiple access (NOMA) systems by optimizing IRS phase shifts [12]. Le-Thanh et al. studied the performance of IRS-assisted cognitive radio systems under different interference levels [13], while Zhu et al. explored the application of IRS in heterogeneous cognitive radio networks [14].

Despite the rapid progress in IRS-assisted cognitive radio networks, several critical issues remain insufficiently addressed. Most existing studies focus on optimizing either IRS phase shifts or transmit power allocation in isolation, while the joint optimization of IRS configurations and NOMA power allocation under practical energy-harvesting constraints is still limited. Moreover, many works assume energy-unconstrained relays and neglect fairness among secondary users, which may lead to uneven resource allocation. In addition, conventional convex optimization-based approaches may become computationally inefficient or prone to local optima when dealing with high-dimensional IRS phase variables. These gaps motivate the present work.

In this paper, an IRS-assisted energy-constrained relay cognitive radio network is developed, with the objective of maximizing the energy efficiency of the CR network under energy-harvesting and power constraints. To this end, the IRS phase shift matrix and power allocation coefficients are jointly modeled and treated as optimization variables. However, the joint optimization of IRS phase shifts and power allocation is highly non-convex, tightly coupled, and high-dimensional, making direct optimization computationally intractable. To overcome these challenges, a chaotic spider wasp optimization (CSWO) algorithm is employed to solve the proposed joint optimization problem. The spider wasp optimization (SWO) algorithm is a recently developed intelligent optimization technique characterized by fast convergence speed and high solution accuracy [15]. By incorporating chaotic mechanisms, population diversity and global exploration capability are further enhanced, effectively reducing the risk of premature convergence and improving overall convergence performance.

The main contributions of this paper are summarized as follows:

(1) We develop an IRS-assisted energy-harvesting relay-based cognitive radio network operating in the underlay mode, where the relay has no external power supply and relies solely on harvested RF energy. By jointly exploiting controllable reflective links and cooperative relaying, the proposed model effectively mitigates severe path loss and enhances energy utilization efficiency.
(2) By integrating NOMA with SIC at the secondary users, the proposed framework improves spectrum utilization and supports multiple secondary users simultaneously under strict interference constraints imposed on the primary user.
(3) We formulate a non-convex energy efficiency maximization problem that jointly optimizes the IRS phase shifts and NOMA power allocation coefficients, subject to interference, energy causality, and QoS constraints. This formulation provides a comprehensive characterization of the trade-offs between energy efficiency and interference management in IRS-assisted CRNs.
(4) To efficiently solve the highly non-convex problem, we propose a chaotic Spider Wasp Optimization (CSWO) algorithm, which enhances population diversity and global exploration capability compared with conventional SWO and GWO. Simulation results demonstrate that the proposed CSWO achieves superior energy efficiency and faster convergence.

## 2. System Model

**Figure 1:**
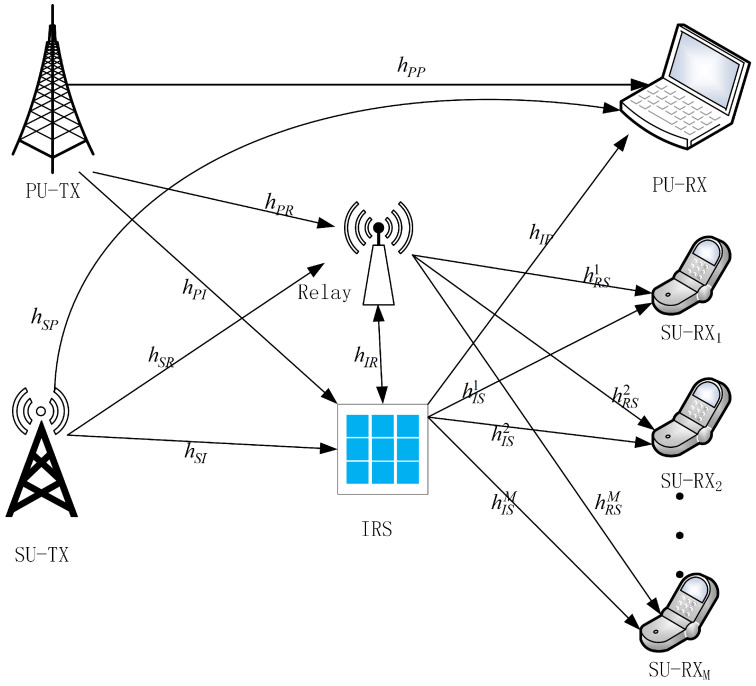
IRS-assisted energy-harvesting relay-based underlay cognitive radio network model.

This paper investigates a cognitive radio relay communication system comprising both primary users (PU) and secondary users (SU), as shown in Figure 1.

The considered system consists of one primary user transmitter (PU−TX), one primary user receiver (PU−RX), one secondary user transmitter (SU−TX), multiple secondary user receivers denoted by $SU-{Rx}_{m}\left(m=1,2,\ldots M\right)$, and an energy-constrained relay equipped with an amplify-and-forward (AF) protocol. To improve link quality and enhance controllable reflection gains, an intelligent reflecting surface (IRS) with *N* reflecting elements is deployed. The IRS reflection coefficient matrix is denoted by $\Theta =diag\left(e^{j\Theta_{1}},e^{j\Theta_{2}},\ldots e^{j\Theta_{N}}\right)$, where $\theta_{n}$ represents the phase shift of the nth reflecting element. We assume that the IRS elements can adjust their phase shifts continuously within $\left[0,2\pi \right]$. This assumption is commonly used to characterize the upper-bound performance of IRS-assisted systems [16,17]. Let $h_{PP}$ denote the channel gain between PU−TX and PU−RX. The channel gains from PU−TX and SU−TX to the relay are denoted by $h_{PR}$ and $h_{SR}$, respectively, while $h_{SP}$ represents the channel gain between SU−TX and PU−RX. Furthermore, $h_{PI}$ and $h_{SI}$ denote the channel gains from PU−TX and SU−TX to the IRS, respectively, $h_{IR}$ denotes the channel gain between the IRS and the relay, $h_{IP}$ represents the channel gain from the IRS to PU−RX, $h_{RS}^{i}$ denotes the channel gain from the relay to the ith SU−RX, and $h_{IS}^{j}$ represents the channel gain from the IRS to the jth SU−RX.

The relay in the considered system is an energy-constrained device without a continuous external power supply. Instead, it harvests energy from the radio-frequency (RF) signals transmitted by PU−TX and SU−TX. The transmission process is divided into two equal time phases. In the first phase (the first 1/2T, where *T* represents the entire time period for signal transmission), the relay performs energy harvesting by collecting energy from the uplink signals of both the PU and SU, where the signals reflected by the IRS further enhance the incident energy. In the second phase (last 1/2T), the relay operates in amplify-and-forward mode, amplifying the received superimposed signal and forwarding it to PU−RX and $SU-{Rx}_{m}$.

To improve spectrum utilization efficiency and enable multi-user access for secondary users while sharing the licensed spectrum of the primary user, non-orthogonal multiple access (NOMA) is adopted at the secondary user side. Specifically, the SU−TX transmits superimposed signals to multiple secondary users simultaneously over the same time–frequency resource, and power allocation is performed according to the ordering of users’ channel gains. Assume that the system consists of *M* secondary users, whose channel gains are ordered as:

(1) $${\left|h_{RS}^{1}\right|}^{2}\geq {\left|h_{RS}^{2}\right|}^{2}\geq \cdots \geq {\left|h_{RS}^{M}\right|}^{2}$$

For SU *m*, SU−TX allocates a power coefficient for it, which satisfies:

(2) $$\sum_{i=1}^{M}\alpha_{m}\leq 1,\;\alpha_{1}\leq \alpha_{2}\leq \cdots \leq \alpha_{M}$$

In the first stage, PU−TX transmits the signal to PU−RX. The signal received by the primary user receiver can be expressed as:

(3) $$y_{PU}^{\left(1\right)}=\sqrt{P_{T}}\left(h_{PP}+h_{PI}\Theta^{\left(1\right)}h_{IP}\right)x_{p}+\sum_{k=1}^{M}\sqrt{\alpha_{k}P_{S}}\left(h_{SP}+h_{SI}\Theta^{\left(1\right)}h_{IP}\right)x_{s,k}+n_{PU}^{\left(1\right)}$$

where $P_{T}$ represents the transmit power of the PU−TX, $x_{P}$ and $x_{s,k}$ represent the message sent by PU−TX and SU−TX, respectively, $P_{S}$ represents the transmit power of SU, $\Theta^{1}$ represents the phase matrix of the first stage of IRS, and $n_{PU}^{1}∼CN\left(0,\sigma_{k}^{2}\right)$ indicates the additive Gaussian white noise at PU.

In this phase, the signal received at relay can be expressed as:

(4) $$y_{R}^{\left(1\right)}=\sum_{k=1}^{M}\sqrt{\alpha_{k}P_{S}}\left(h_{SR}+h_{SI}\Theta^{\left(1\right)}h_{IR}\right)x_{s,k}+\sqrt{P_{T}}\left(h_{PR}+h_{PI}\Theta^{\left(1\right)}h_{IR}\right)x_{p}+n_{R}^{1}$$

where $n_{R}^{1}∼CN\left(0,\sigma_{k}^{2}\right)$ represents the additive Gaussian white noise at relay.

Since the relay does not have an external power supply, it performs energy collection at this stage. The energy collected during this stage can be expressed as:

(5) $$E_{P}=\frac{1}{2}T\eta \delta {\left|y_{R}^{\left(1\right)}\right|}^{2}$$

where *T* represents the total time of the entire transmission stage. $\eta \in \left(0,1\right)$ represents energy harvesting efficiency and $\delta \in \left(0,1\right)$ represents the proportion of signal power used for energy harvest.

In the second phase, the relay utilizes the energy harvested in the first phase to forward the signal. Accordingly, the transmit power of the relay can be expressed as:

(6) $$P_{R}=\frac{E_{P}}{(1/2)T}=\eta \delta {\left|y_{R}^{\left(1\right)}\right|}^{2}$$

To enhance the communication quality of the wireless channel, the relay amplifies the received signal before forwarding it. Accordingly, the signal transmitted after relay amplification can be expressed as:

(7) $$x_{R}=A\sqrt{1-\delta }\sqrt{P_{R}}y_{R}^{\left(1\right)}$$

where *A* denotes the amplification factor, which is calculated as follows:

(8) $$\begin{array}{cc} A & =\sqrt{\frac{1}{\left(1-\delta \right)\left({\left|y_{R}^{\left(1\right)}\right|}^{2}+\sigma_{R}^{2}\right)}}\\ \; & \approx \sqrt{\frac{1}{\left(1-\delta \right){\left|y_{R}^{\left(1\right)}\right|}^{2}}} \end{array}$$

By substituting it into Equation (7), the signal forwarded by the relay can be expressed as $x_{R}=\sqrt{\eta \delta }y_{R}^{\left(1\right)}$. The relay then directly transmits this signal to the SU−RX nodes. Accordingly, the signal received at the mth SU−RX can be expressed as follows:

(9) $$y_{m}^{\left(2\right)}=h_{RS}^{m}x_{R}+h_{IS}^{m}\Theta^{\left(2\right)}h_{IR}x_{R}+n_{m}^{\left(2\right)}$$

where $n_{m}^{\left(2\right)}$ denotes the background noise amplified by the relay. By substituting into Equation (9), the above expression can be expanded as follows:

(10) $$y_{m}^{\left(2\right)}=\sqrt{\eta \delta }\left(h_{RS}^{m}+h_{IS}^{m}\Theta^{\left(2\right)}h_{IR}\right)y_{R}^{\left(1\right)}+n_{m}^{\left(2\right)}$$

The received signal at each user still contains superimposed interference from other users. Therefore, successive interference cancellation (SIC) is employed at the receiver to perform signal decoding. For the mth SU, the decoding order follows the NOMA principle: the signals of users with weaker channel gains than that of user *m* are first detected and canceled, whereas the signals of users with stronger channel gains are treated as non-cancelable interference. Accordingly, the signal-to-interference-plus-noise ratio (SINR) of the mth secondary user can be expressed as:

(11) $$\gamma_{m}=\frac{\eta \delta \alpha_{m}P_{S}{\left|H_{m}\right|}^{2}}{\eta \delta \left(\sum_{j=m+1}^{K}\alpha_{j}P_{S}{\left|H_{m}\right|}^{2}+P_{T}{\left|G_{m}\right|}^{2}+\sigma_{R}^{2}{\left|R_{m}\right|}^{2}\right)+\sigma_{m}^{2}}$$

where $H_{m}=\left(h_{RS}^{m}+h_{IS}^{m}\Theta^{\left(2\right)}h_{IR}\right)\left(h_{SR}+h_{SI}\Theta^{\left(1\right)}h_{IR}\right)$ denotes the composite channel gain of the secondary user, $\sigma_{R}^{2}$ represents the noise power at the relay, ${\left|\begin{array}{c} G_{m} \end{array}\right|}^{2}={\left|\begin{array}{c} \left(h_{RS}^{m}+h_{IS}^{m}\Theta^{\left(2\right)}h_{IR}\right)\left(h_{PR}+h_{PI}\Theta^{\left(1\right)}h_{IR}\right) \end{array}\right|}^{2}$ denotes the composite channel gain corresponding to the interference from the primary user, ${\left|R_{m}\right|}^{2}={\left|h_{RS}^{m}+h_{IS}^{m}\Theta^{\left(2\right)}h_{IR}\right|}^{2}$ represents the amplified interference channel gain and $\sigma_{m}^{2}$ denotes the noise power at the secondary user receiver.

Our objective is to maximize the energy efficiency of the system. The energy efficiency, defined as the ratio of the system throughput to the total power consumption of the system, can be shown as:

(12) $$EE=\frac{R_{sum}}{P_{total}}$$

where $R_{sum}$ denotes the total system throughput, $P_{total}$ represents the total energy consumption of the system, and the achievable rate of the mth SU is given by:

(13) $$R_{m}=\frac{1}{2}\log_{2}\left(1+\gamma_{m}\right)$$

Therefore, the system throughput can be expressed as:

(14) $$R_{sum}=\sum_{m=1}^{M}R_{m}=\sum_{m=1}^{M}\frac{1}{2}\log_{2}\left(1+\gamma_{m}\right)$$

The power consumption in the considered system mainly consists of the following components: (1) the transmit power of the secondary user transmitter; (2) the forwarding power of the relay. In addition, the IRS itself is assumed to consume no power, and the power consumption of the control circuitry is neglected; (3) the fixed circuit power consumption, which is assumed to be a constant. Accordingly, the total power consumption of the system can be expressed as:

(15) $$P_{total}=P_{S}+P_{R}+P_{C}$$

Therefore, our optimization objective can be expanded as:

(16) $$\max EE=\frac{1}{2}\cdot \frac{\sum_{m=1}^{M}\log_{2}\left(1+\gamma_{m}\right)}{P_{S}+P_{R}+P_{C}}$$

To guarantee the communication quality of the primary user, the instantaneous interference power caused by the secondary user and the relay at the PU-RX must not exceed the maximum interference threshold $I_{th}$ tolerable by the primary user at any time.

(17) $$P_{S}{\left|h_{SP}+h_{SI}\Theta^{\left(1\right)}h_{IP}\right|}^{2}+P_{R}\left|h_{IR}\Theta^{\left(2\right)}h_{IP}\right|\leq I_{th}$$

Meanwhile, to guarantee the communication quality among secondary users, the SINR of each secondary user is required to be no lower than a predefined minimum threshold $\gamma_{min}$.

(18) $$\gamma_{m}\geq \gamma_{\min}$$

Considering the different channel conditions among different secondary users in the NOMA scenario, to prevent excessive resource allocation to a subset of users, fairness in power allocation among secondary users must be ensured. In this paper, Jain’s fairness index is adopted as the performance metric, which is defined as:

(19) $$J\left(\alpha \right)={\left(\sum_{m=1}^{M}\alpha_{m}\right)}^{2}/M\sum_{m=1}^{M}\alpha_{m}^{2}$$

where $\alpha_{m}$ denotes the power allocation coefficient assigned to the mth secondary user. A larger value of Jain’s fairness index indicates a more equitable resource allocation among secondary users.

Under the above constraints, our optimization objective can be further expressed as:

(20) $$\begin{array}{c} \max\;EE=\frac{1}{2}\cdot \frac{\sum_{m=1}^{M}\log_{2}\left(1+\gamma_{m}\right)}{P_{S}+P_{R}+P_{C}}\\ C1:\sum_{i=1}^{M}\alpha_{m}\leq 1,\;\alpha_{1}\leq \alpha_{2}\leq \cdots \leq \alpha_{M}\\ C2:0<\alpha_{m}<1,\;\forall \alpha_{m}\\ C3:P_{S}{\left|h_{SP}+h_{SI}\Theta^{\left(1\right)}h_{IP}\right|}^{2}+P_{R}\left|h_{IR}\Theta^{\left(2\right)}h_{IP}\right|\leq I_{th}\\ C4:\gamma_{m}\geq \gamma_{\min}\\ C5:J\left(\alpha \right)\geq J_{\min}\left(\alpha \right) \end{array}$$

In this paper, a hybrid channel fading model is adopted. Specifically, Rician fading is employed to characterize all IRS-related links due to the existence of dominant line-of-sight (LoS) components enabled by the intelligently deployed reflecting surface [18], e.g., $h_{SI},\;h_{PI},\;h_{IR}$, while Nakagami-m fading is used to model the non-IRS direct links, which are subject to rich scattering and non-line-of-sight (NLoS) propagation conditions [19]. e.g., $h_{SR},\;h_{PR},\;h_{SP}$. The links related to the IRS are modeled as

(21) $$h_{XY}^{\mathrm{IRS}}=\sqrt{\frac{K_{XY}}{K_{XY}+1}}h_{XY}^{\mathrm{LoS}}+\sqrt{\frac{1}{K_{XY}+1}}h_{XY}^{\mathrm{NLoS}}$$

where $K_{XY}$ is the Rician factor, $h_{XY}^{LoS}$ and $h_{XY}^{NLoS}$ are the corresponding line-of-sight(LoS) channel matrix and non-line-sight(NLoS) channel matrix, respectively.

The channel model for non-IRS direct-link communication is modeled as

(22) $$h_{XY}^{LoS}{\left|h_{XY}\right|}^{2}∼\mathrm{Gamma}\left(m_{XY},\Omega_{XY}\right)$$

The commonly used probability density function (pdf) for Nakagami-m fading can be given by

(23) $$f_{{\left|h\right|}^{2}}\left(x\right)=\frac{m^{m}}{\Gamma \left(m\right)\Omega^{m}}x^{m-1}\exp\left(-\frac{mx}{\Omega }\right)$$

where $x={\left|h\right|}^{2}$ denotes the channel power gains, $\Omega =E\left[x\right]$ is the average power, *m* represents the fading severity parameter.

## 3. Chaotic SWO Algorithm for Power Allocation

The Spider Wasp Optimization (SWO) algorithm is a novel intelligent optimization method that simulates the hunting, nesting, and mating behaviors of female spider wasps to obtain optimal solutions, offering advantages such as fast convergence and high solution accuracy. However, when dealing with complex optimization problems, it may still fall into local optima. To address this issue, we introduce chaotic theory to enhance the algorithm’s performance.

### 3.1. Logistic-Tent Chaotic Map

In conventional intelligent optimization algorithms, the rand function is typically used to randomly generate the population. However, populations generated in this way often suffer from uneven distribution. Chaotic maps, due to their good randomness and global exploration properties, can effectively address this issue. In this work, we adopt the Logistic-Tent chaotic map, whose mapping principle is as follows:

(24) $$x_{n+1}=\left\{\begin{array}{c} \left[rx_{n}\left(1-x_{n}+\frac{4-r}{2}x_{n}\right)mod1\right],x_{n}<0.5\\ \left[rx_{n}\left(1-x_{n}\right)+\frac{\left(4-r\right)\left(1-x_{n}\right)}{2}\right]mod1,x_{n}\geq 0.5 \end{array}\right.$$

where $x_{n}$ and $x_{n+1}$ represent the states at the nth and n+1th iterations, respectively, and $r\in \left(0,4\right)$ is the control parameter used to regulate the chaos of the map and he larger the value, the higher the degree of chaos. By introducing the chaotic map, the population can be distributed more uniformly, which helps the algorithm better explore the solution space and avoid premature convergence to local optima.

In this work, we not only use the chaotic map to generate the initial population but also apply chaotic perturbations when the algorithm gets trapped in a local optimum to help it escape. The condition we use to determine that the algorithm has fallen into a local optimum is:

(25) $$\left|f_{best}\left(G\right)-f_{best}\left(t-G\right)\right|<\varepsilon$$

where $f_{best}\left(G\right)$ denotes the best fitness value of the individuals in the ith generation, and ε represents the threshold for triggering the chaotic map. When the algorithm shows no significant improvement over *G* generations, the chaotic map is activated, and the magnitude of the perturbation is described by the following formula:

(26) $$\Delta \left(t\right)=\Delta_{0}{\left(1-\frac{t}{T_{\max}}\right)}^{\gamma }$$

where $\Delta_{0}$ indicates the range of variation of the variable and $\gamma \in \left(1,2\right)$.

The update of individuals after the chaotic mapping can be expressed by the following formula:

(27) $$X_{i}^{\prime }\left(t\right)=X_{i}\left(t\right)+\Delta \left(t\right)\left(2c_{t}-1\right)$$

where $2c_{t}-1\in \left(-1,1\right)$ denotes the random direction of the chaotic perturbation, and $\Delta \left(t\right)$ represents the perturbation magnitude, which decreases as the number of iterations increases. If the new solution obtained from the chaotic map is better than the previous one, it is retained; otherwise, no action is taken.

### 3.2. Spider Wasp Optimization Algorithm

In hunting and nesting behavior, the position change of an individual is determined by the following formula:

(28) $$x_{i}^{t+1}=\left\{\begin{array}{c} \begin{array}{ccc} x_{i}^{t}+\left|m\right|*\delta_{ab} & \; & i<K;p<k;r_{1}<r_{2}\\ x_{c}^{t}+B*\left(L+Q\right) & \; & i<K;p<k;r_{1}\geq r_{2}\\ x_{i}^{t}+\lambda *r_{3}*\left|2*\delta_{ci}\right| & \; & i<K;p\geq k;r_{1}<r_{2}\\ x_{i}^{t}*\nu c & \; & i<K;p\geq k;r_{1}\geq r_{2}\\ x^{*}+\mu_{1}*\left(x^{*}-x_{i}^{t}\right) & \; & i\geq K;r_{1}<r_{2}\\ x_{a}^{t}+\left|\gamma \right|*\delta_{ai}+A*U*\mu_{bc} & \; & i\geq K;r_{1}\geq r_{2} \end{array} \end{array}\right.$$

where $x_{i}^{t}$ and $x_{i}^{t+1}$ represent the individuals in the population at time *t* and time t+1, respectively, K=N∗k, *N* represents the number of population, $k=1-t/t_{\max}$, $r_{1},\;r_{2},\;r_{3},\;p,\;k$ is a random number between 0 and 1, and rn is a random number following a normal distribution. $\delta_{mn}=r*\mu_{mn}$, $r\in \left[0,1\right]$ is a random number. $\mu_{mn}=x_{m}^{t}-x_{n}^{t}$, $\mu_{1}=\cos\left(2\pi l\right)$, $B=\mu_{1}/1+e^{l}$, where *l* denotes a random number between −2 and 1, Q=r∗(H−L), and *H* and *L* represent the upper and lower bounds of the search space, respectively. $\lambda =2-2*t/t_{\max}$, vc denotes a rand number between −k and *k*, $x^{*}$ represents the best solution at present, A=1−r, γ is a random number generated by the Levy distribution, and *U* is calculated as follows:

(29) $$U=\left\{\begin{array}{cc} 1, & r_{4}\geq r_{5}\\ 0, & r_{4}<r_{5} \end{array}\right.$$

In the mating behavior, new individuals are generated according to the following formula:

(30) $$x_{m}^{t+1}=x_{i}^{t}+e^{l}*\left|\begin{array}{c} \beta \end{array}\right|*v_{1}+\left(\begin{array}{c} 1-e^{l} \end{array}\right)*\left|\begin{array}{c} \beta_{1} \end{array}\right|*v_{2}$$

where β and $\beta_{1}$ are two numbers generated according to the normal distribution. $v_{1}$ and $v_{2}$ are determined by the following formula:

(31) $$v_{1}=\left\{\begin{array}{cc} x_{a}-x_{i}, & f\left(x_{a}\right)<f\left(x_{i}\right)\\ x_{i}-x_{a}, & f\left(x_{a}\right)\geq f\left(x_{i}\right) \end{array}\right.$$

(32) $$v_{2}=\left\{\begin{array}{cc} x_{b}-x_{c}, & f\left(x_{b}\right)<f\left(x_{c}\right)\\ x_{c}-x_{b}, & f\left(x_{b}\right)\geq f\left(x_{c}\right) \end{array}\right.$$

where $x_{a}$, $x_{b}$ and $x_{c}$ are individuals randomly selected from the entire population. $x_{i}$ represent the current individual, $f\left(*\right)$ represents the current fitness.

To accelerate the algorithm’s convergence, the population size is updated according to the following formula:

(33) $$N=N_{\min}+\left(N-N_{\min}\right)\times k$$

where $N_{\min}$ represents the minimum number of the population.

The trade-off between hunting, nesting behavior and mating behavior is controlled by a preset value TR. However, to improve the balance between exploration and exploitation during the optimization process, a dynamic trade-off factor TR is introduced to adaptively regulate the probabilities of hunting, nesting, and mating behaviors in the CSWO algorithm. Specifically, a larger value of TR encourages hunting and nesting behaviors, which enhances global exploration and helps the algorithm escape from local optima in the early stages. As the iteration progresses, a smaller TR increases the likelihood of mating behavior, thereby strengthening local exploitation and accelerating convergence toward high-quality solutions. Therefore, we improve the calculation of TR as follows:

(34) $$TR\left(t\right)={TR}_{\min}+\frac{\left({TR}_{\max}-{TR}_{\min}\right)\left(1+\cos\left(\pi t/T_{\max}\right)\right)}{2}$$

where ${TR}_{min}$ represents the minimum value of TR and ${TR}_{max}$ represents the maximum value of TR.

**Figure 2:**
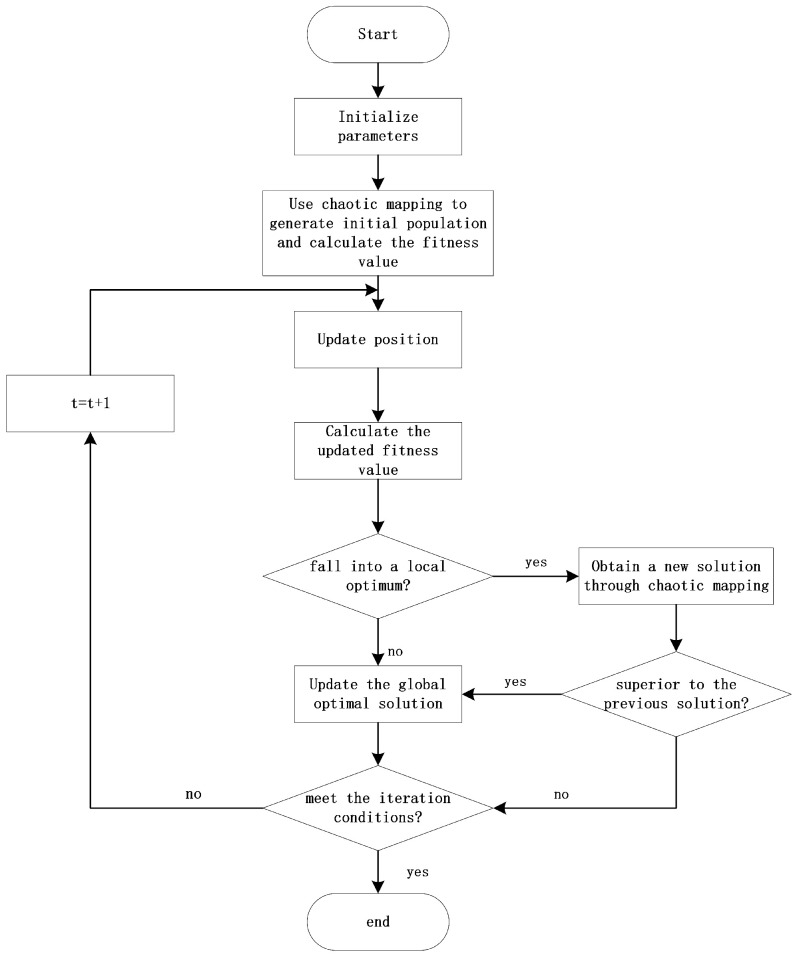
Algorithm flowchart.

The algorithm flow is illustrated in Figure 2:

## 4. Result Analysis

In this section, simulation experiments are conducted to verify the performance of the IRS-based chaotic Spider Wasp Optimization algorithm. In the experiments, part of the parameter settings are specified as follows: the number of PUs is set to 1, the number of SUs is set to 4, and the number of IRS elements is set to 64. The maximum interference that the PU can tolerate $I_{th}$ is set to 0.15 mW. The minimum SINR requirement $\gamma_{min}$ is set to 2 dB, and the minimum fairness requirement $J_{min}\left(\alpha \right)$ is set to 0.9. The transmit power of PU-TX is set to 2 W. All simulation results are obtained by averaging over multiple independent Monte Carlo runs under random channel realizations to ensure statistical reliability.

### 4.1. Time Complexity Analysis

In this section, we analyzed the time complexity of the chaotic Spider Wasp Optimization (CSWO), Spider Wasp Optimization (SWO), Grey Wolf Optimization (GWO), and Particle Swarm Optimization (PSO). The computational complexity of these algorithm is mainly determined by the population size *N*, the problem dimension *M* and the maximum number of iterations *T*. The following are the time complexity of these algorithms.

(35) $$\begin{array}{cc} \; & O\left(CSWO\right)=O\left(T\cdot N\cdot M\right)\\ \; & O\left(SWO\right)=O\left(T\cdot N\cdot M\right)\\ \; & O\left(PSO\right)=O\left(T\cdot N\cdot M\right)\\ \; & O\left(GWO\right)=O\left(T\cdot \left(NlogN+NM\right)\right) \end{array}$$

Based on the above analysis of the time complexity of the algorithms, we compared the running times and average running times of all the algorithms, as shown in Table 1.

### 4.2. Simulation Analysis

**Figure 3:**
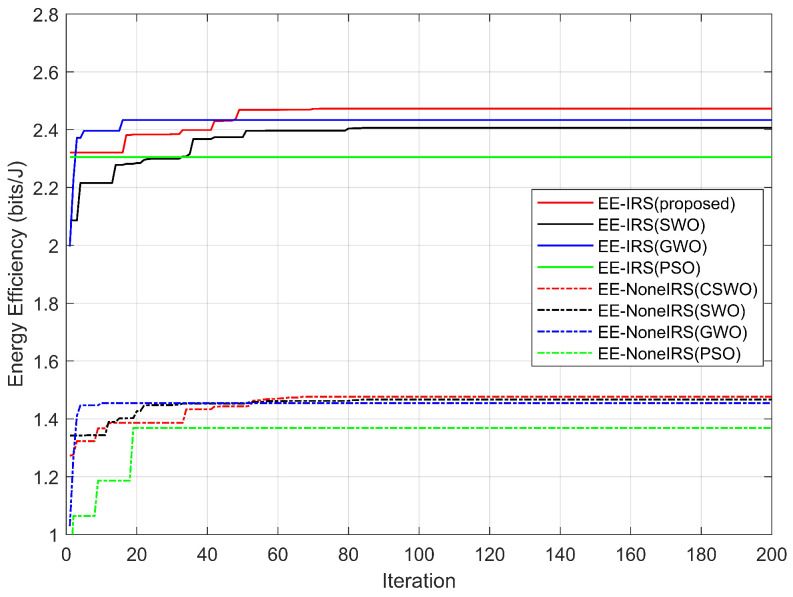
Energy efficiency comparison under different algorithms using IRS and without using IRS.

Figure 3 illustrates the convergence behavior and energy efficiency performance of different optimization algorithms, including the proposed CSWO, SWO, GWO, and PSO, under identical system settings, with and without the assistance of an intelligent reflecting surface (IRS). As observed from the figure, the IRS-assisted schemes consistently achieve higher energy efficiency than the corresponding non-IRS schemes for all considered algorithms. This performance improvement validates the effectiveness of IRS in enhancing energy efficiency by exploiting cascaded channel gains and improving signal propagation conditions. Moreover, all algorithms exhibit stable convergence behaviors in both scenarios, indicating their feasibility in solving the formulated non-convex optimization problem. Among the compared methods, the proposed CSWO algorithm achieves the highest final energy efficiency and demonstrates faster convergence compared with SWO, GWO, and PSO. This superiority is evident in both IRS-assisted and non-IRS cases, highlighting the robustness of the proposed algorithm. The performance gain of CSWO mainly stems from the incorporation of chaotic strategies, which enhance population diversity in the early stages and improve the balance between global exploration and local exploitation, thereby reducing the risk of premature convergence. Overall, the results confirm that IRS deployment significantly improves network energy efficiency, while the proposed CSWO algorithm further enhances optimization performance, demonstrating the effectiveness of the proposed system model and optimization framework.

**Figure 4:**
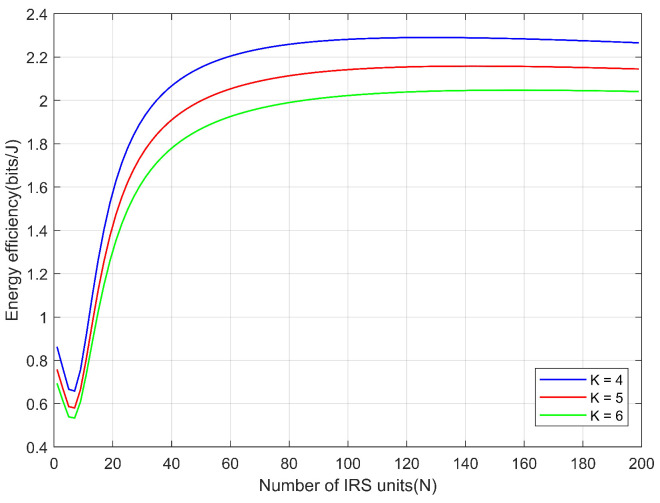
The impact of the number of IRS units on energy efficiency under different users K.

Figure 4 illustrates the impact of the number of IRS elements on the system energy efficiency under different numbers of users. The results show that as the number of IRS elements increases, the energy efficiency first decreases and then rises rapidly, reaching an inflection point around a certain value. After that, the growth of energy efficiency gradually becomes saturated. This is because the cascaded gain provided by the IRS tends to stabilize when a large-scale reflecting array is deployed. For different user numbers, the corresponding curves exhibit a similar variation trend. However, as the number of users increases, the total interference power consumption of the system becomes larger, which leads to an overall reduction in energy efficiency. These results indicate that appropriately increasing the number of IRS elements can significantly enhance system energy efficiency, whereas under large-scale IRS deployment, the marginal energy efficiency gain gradually.

**Figure 5:**
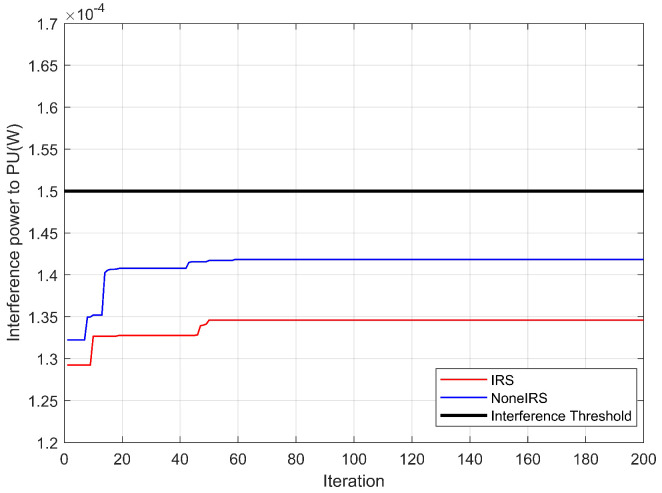
Comparison of interference power imposed on the primary user with and without IRS.

Figure 5 compares the interference imposed on the primary user by secondary user transmissions when the chaotic Spider Wasp Optimization algorithm is applied, with and without IRS. It can be observed that the interference power in the IRS-assisted case is higher than that in the non-IRS case. This is because the IRS not only reflects the desired signals from the secondary users but also redirects part of the interference signals toward the primary user receiver. Nevertheless, the interference power in both the IRS-assisted and non-IRS scenarios remains below the maximum interference threshold tolerated by the primary user. This result demonstrates that the proposed algorithm does not compromise the normal communication of the primary user.

**Figure 6:**
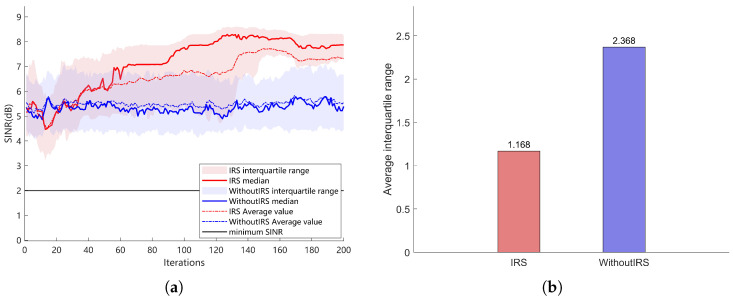
SINR comparison under different scenarios. (**a**) Comparison of SINR with and without IRS. (**b**) Average interquartile range with and without IRS.

Figure 6a illustrates the variation of the signal-to-interference-plus-noise ratio (SINR) obtained by the chaotic Spider Wasp Optimization algorithm in scenarios with and without IRS. It can be observed that both the median and the average SINR in the IRS-assisted case are higher than those in the non-IRS case, and the curve exhibits a continuously increasing trend. This indicates that IRS can effectively enhance the cascaded channel gain, thereby significantly improving the communication quality of the system. In contrast, in the absence of IRS, the SINR remains at a relatively low level with larger fluctuations. Figure 6b presents a comparison of the average interquartile range (IQR) over the last 20 iterations for the two scenarios. It can be seen that the average IQR with IRS is significantly smaller than that without IRS, which further demonstrates that the use of IRS not only yields higher SINR gains but also improves the stability of the system. Overall, IRS shows clear advantages in enhancing signal quality and reducing SINR fluctuations, validating the effectiveness of the proposed system model.

**Figure 7:**
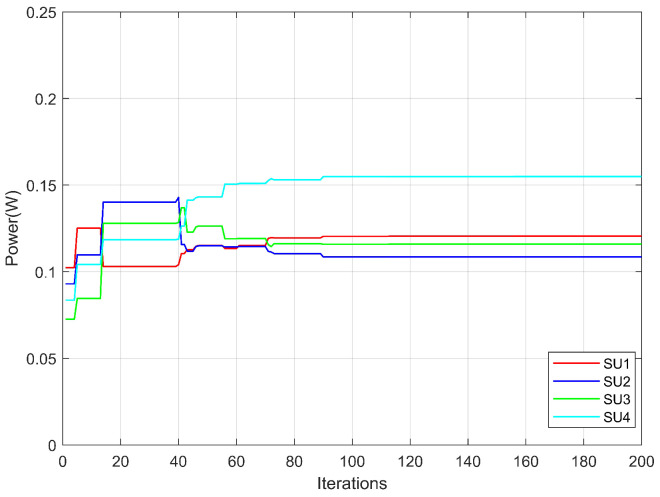
Power allocation of each secondary user.

Figure 7 illustrates the convergence process of power allocation for each secondary user over the iterations. It can be observed that the power allocation gradually stabilizes after approximately 80 iterations, indicating that the algorithm has reached a good convergence state at this stage. Moreover, the power allocation curves for all secondary users are smooth and uniform, with no bias toward any particular user, demonstrating a high degree of fairness in power distribution. These results validate the effectiveness of the fairness constraint introduced in this work, ensuring that all secondary users can achieve stable and reasonable communication performance. Consequently, each user is able to communicate efficiently, highlighting the necessity of the proposed fairness constraint.

**Figure 8:**
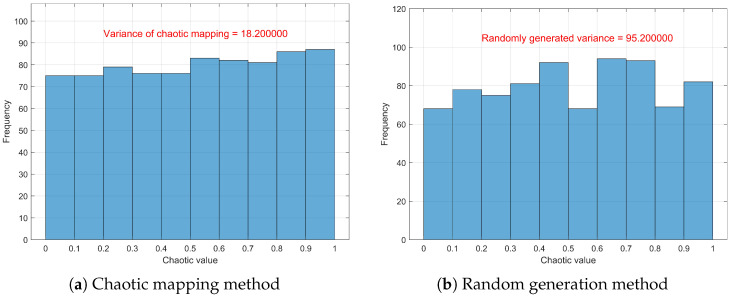
Comparison between chaotic mapping and random generation methods.

Figure 8a illustrates the frequency distribution of the initial population generated based on chaotic mapping, while Figure 8b shows the frequency distribution of the population generated using random initialization. It can be observed that the population generated by chaotic mapping is more evenly distributed across different regions, with smaller frequency variance, which facilitates a more comprehensive exploration of the solution space in the initial stage and enhances the global search capability of the algorithm. In contrast, individuals generated through random initialization are relatively scattered and tend to cluster in certain regions during the early stage, increasing the risk of falling into local optima. These results validate the rationality and necessity of constructing the initial population using chaotic mapping.

## 5. Conclusions

This paper employs the chaotic Spider Wasp Optimization (CSWO) algorithm to jointly optimize IRS phase shifts and power allocation coefficients, aiming to maximize system energy efficiency under the underlay mode. The constructed signal transmission model can significantly enhance energy efficiency, while the proposed improved optimization algorithm effectively overcomes the tendency of traditional methods to get trapped in local optima, demonstrating stronger global search capability. To validate the effectiveness of the approach, comparisons were conducted between the energy efficiency achieved with and without IRS under different algorithms, and further comparisons were made for the same algorithm under the two model conditions. Simulation results indicate that the proposed model and algorithm not only achieve higher energy efficiency but also exhibit better stability and robustness.

## Figures and Tables

**Table 1:** Comparison of running time for different algorithms.

Algorithms	First	Second	Third	Fourth	Fifth	Average Running Time (s)
CSWO	4.869	4.734	4.577	4.559	4.689	4.7076
SWO	4.401	4.342	4.167	4.129	4.406	4.289
GWO	6.632	6.741	6.854	6.129	6.677	6.6066
PSO	3.709	3.809	4.134	3.903	3.852	3.881

## Data Availability

The original contributions presented in this study are included in the article. Further inquiries can be directed to the corresponding author.
